# High-fat diet-induced obesity accelerates puberty in male rats through SMIM20/phoenixin upregulation

**DOI:** 10.3389/fendo.2025.1711374

**Published:** 2025-11-21

**Authors:** Tao Xie, Wei Qin, Dan Zeng, Runqi Wang, Shuting Chen, Yanfei Chen, Jingzi Zhong, Dan Lan

**Affiliations:** 1 Department of Pediatrics, The First Affiliated Hospital of Guangxi Medical University, Nanning, China; 2 Difficult and Critical Illness Center, Pediatric Clinical Medical Research Center of Guangxi, Nanning, China; 3 The Key Laboratory of Children’s Disease Research in Guangxi’s Colleges and Universities, Education Department of Guangxi Zhuang Autonomous Region, Nanning, China; 4 Guangxi Medical University, Nanning, China; 5 The First People’s Hospital of Nanning, Nanning, China

**Keywords:** phoenixin, high-fat diet, early puberty, obesity, male rats

## Abstract

**Background:**

Controversy exists regarding the relationship between obesity and pubertal onset in boys, and the underlying mechanisms remain unclear.

**Objective:**

To establish a high-fat diet (HFD)-induced obesity model in juvenile male Sprague-Dawley (SD) rats, and to investigate the relationship between obesity and pubertal advancement as well as the role of *Smim20*/phoenixin (PNX) in male pubertal development.

**Methods:**

A HFD (45% fat) was administered to male SD rats to induce obesity, while control rats were maintained on a normal diet (ND) from birth. Body weight and preputial separation were monitored as markers of obesity and pubertal onset. At prepubertal (postnatal day 30, PND30) and early pubertal (PND40) stages, serum, hypothalamus, pituitary, testes, and adipose tissue were collected. RT-qPCR was performed to measure the mRNA expression levels of key genes in the hypothalamic–pituitary–gonadal axis (HPGA), including gonadotropin-releasing hormone (*GnRH*), *Kiss1*, G protein-coupled receptor 54 (*GPR54*), GnRH receptor (*GnRHr*), and *Smim20*. Serum luteinizing hormone (LH), follicle-stimulating hormone (FSH), testosterone, and PNX protein were measured by ELISA. Associations among obesity (body mass index, BMI), PNX, and pubertal timing were evaluated using Spearman’s correlation.

**Results:**

HFD-fed rats exhibited significantly greater body weight and fat mass than ND-fed rats at both time points. (P<0.001), with earlier preputial separation (P<0.001). Testicular weight and expression of *GnRH, Kiss1, GPR54, and GnRH*r were increased, alongside higher serum LH, FSH, and testosterone (all P<0.05). PNX expression in hypothalamus, pituitary, testes, and subcutaneous fat, as well as serum PNX-14 and PNX-20 levels, were significantly elevated in HFD rats compared with controls (P<0.05). After adjusting for BMI, serum PNX-20 and PNX-14 (P<0.001) remained higher in the HFD group. Body weight was negatively correlated with age at preputial separation and positively correlated with serum LH, testosterone, abdominal circumference, PNX.

**Conclusion:**

To our knowledge, this study established a novel HFD–induced model of prepubertal obesity and central precocious puberty (CPP) in male rats. The findings suggest that obesity may accelerate pubertal onset, and that Smim20/PNX may participate in regulating pubertal development in males.

## 1 Introduction

Central precocious puberty (CPP) is a common pediatric endocrine disorder caused by premature activation of the hypothalamic–pituitary–gonadal axis (HPGA). It is defined in boys as the development of genitalia and secondary sexual characteristics before the age of 9 (1). Over the past few decades, the global incidence of CPP has risen markedly. A Danish national study reported that from 1998 to 2017, CPP incidence increased sixfold in girls and fifteenfold in boys (2, 3). This upward trend parallels the worldwide surge in childhood obesity. Recent studies have reinforced the link between the two—supporting earlier evidence that obesity promotes earlier puberty in girls, while also revealing a previously unclear association between obesity and pubertal timing in boys (4, 5). Since 1980, the global prevalence of obesity has roughly doubled, now affecting nearly 2.5 billion people and posing a serious public health threat (6, 7). Obesity not only increases the risk of chronic diseases in adulthood but may also affect reproductive function and pubertal timing (3, 6). While obesity is consistently associated with earlier puberty in girls (4, 8, 9), findings in boys are conflicting: some studies report an association with earlier puberty (10, 11), others with delay (12, 13), and some find no relationship (14). Although organic lesions are a leading cause of CPP in boys (15), a subset of cases remain unexplained, emphasizing the need for further research on the mechanisms involved.

Pubertal onset depends on activation of the HPGA, and metabolic signals may trigger the pulsatile secretion of gonadotropin-releasing hormone (GnRH) (16). Metabolic factors linked to obesity, including leptin, insulin, ghrelin, and fatty acids, have been widely studied in relation to female puberty (2, 5, 6), but the mechanisms in males are less clear. Animal studies have demonstrated that HFD feeding can reproduce prepubertal obesity and early puberty in female rodents (17). Given the inconsistent findings on the relationship between male obesity and puberty in clinical studies, and the lack of mechanistic studies in male models, further investigation using rodent species with developmental patterns similar to humans is essential. Exploring metabolic signaling pathways may help clarify the complex interactions between obesity and pubertal regulation in boys. yet corresponding models in males remain scarce, limiting insights into the interaction between obesity and pubertal onset in boys.

Phoenixin (PNX), a neuropeptide encoded by the *Smim20* gene, exists in two isoforms, phoenixin-14 (PNX-14) and phoenixin-20 (PNX-20) (18), and is expressed in HPGA-related tissues such as the hypothalamus, pituitary, and ovaries (18–20). Experimental evidence suggests PNX binds to G protein-coupled receptor 173 (GPR173), activating the cyclic adenosine monophosphate (cAMP)/protein kinase A (PKA) pathway, which in turn increases Kiss1 expression and stimulates GnRH release (21, 22). Knockdown of GPR173 blocks GnRH-induced luteinizing hormone (LH) secretion (23, 24), and intracerebroventricular injection of PNX-targeted siRNA in adult female rats reduces anterior pituitary GnRH expression and delays the estrous cycle (19). Peripherally, PNX regulates adipocyte differentiation and energy balance via the cAMP/Epac pathway (18, 25) and promotes follicle growth and estradiol production through cAMP/PKA signaling and CREB phosphorylation (26). Clinically, Serum PNX levels are significantly elevated in girls and boys with CPP and show a positive correlation with pubertal progression (27, 28).

Despite previous advances, the role of PNX in male puberty remains unclear, and the impact of prepubertal obesity on pubertal onset is still debated. Our earlier clinical research found that serum PNX levels were significantly elevated in boys with CPP and were positively correlated with body mass index (BMI) and pubertal progression (27). Building on these findings, this study established an HFD-induced precocious puberty model in male Sprague–Dawley rats. Beginning on postnatal day 1, pups were fed a high-fat diet to induce obesity. We measured body weight, fat content, sex hormone levels, and the expression of key HPGA-related genes (GnRH, Kiss1, GPR54) and Smim20/PNX at prepubertal (PND30) and early pubertal (PND40) stages. The aim was to determine the link between HFD-induced prepubertal obesity and accelerated puberty in male rats and to explore the role of Smim20/PNX in this process.

## 2 Materials and methods

### 2.1 Ethics approval

All animal experiments were approved by the Animal Ethics Committee of Guangxi Medical University (Approval No.: 202209034) and conducted in accordance with the Guide for the Care and Use of Laboratory Animals of the National Institutes of Health.

### 2.2 Animals and diets

Pregnant SPF-grade Sprague–Dawley (SD) rats were obtained from the Animal Experiment Center of Guangxi Medical University (Nanning, China). They were housed at 25 ± 2°C under a 12-hour light/dark cycle, with free access to food and water. On the first day after birth (PND1), male pups were randomly assigned to either a normal diet (ND, n=19) or a high-fat diet (HFD, n=19) group.

Until weaning (PND21), ND pups received standard chow (D12450B; 70% carbohydrate, 20% protein, 10% fat, Beijing Boaigang Biotechnology Co., Ltd), while HFD pups were fed a high-fat diet (D12451; 35% carbohydrate, 20% protein, 45% fat, with lard as the fat source, Beijing Boaigang Biotechnology Co., Ltd). After weaning, pups were separated from dams, housed in groups of 3–4 per cage at equal density, and continued on the same diet. Samples were collected at two stages: prepuberty (PND30, ND n=9, HFD n=9) and early puberty (PND40, ND n=10, HFD n=10).

### 2.3 Monitoring of growth and puberty

Body weight was measured daily from PND21, rats were weighed daily (accurate to 0.01 g) and their abdominal circumference was measured (accurate to 0.1 cm) at 9:00 a.m. Preputial separation was also monitored as a marker of pubertal onset (29). Obesity was considered successfully induced when HFD rats were at least 20% heavier than ND rats at the same time point (30).

### 2.4 Sample collection

At PND30 and PND40, rats were subjected to inhalation anesthesia induction with 3–4% isoflurane (flow rate 0.6–0.8 L/min) Following the loss of consciousness and achievement of a stable anesthetic plane, blood (1–2 mL) was collected from the orbital venous plexus. The blood samples were centrifuged at 3000 × g for 15 min at 4 °C, and the serum was stored at –80 °C. After confirmation of pedal reflex loss (indicating deep anesthesia), a secondary physical method (cervical dislocation) was applied to ensure definitive euthanasia—a protocol consistent with the recommendations for rodents outlined in the American Veterinary Medical Association (AVMA) Guidelines for the Euthanasia of Animals (2020). Death was confirmed by the absence of spontaneous heartbeat and thoracic respiration. The hypothalamus, pituitary, testes, subcutaneous fat, perirenal fat, and perigonadal fat were immediately dissected on ice. Fat depots were weighed to evaluate adiposity. Portions of subcutaneous fat were fixed in 4% paraformaldehyde for 24 h and paraffin-embedded, while remaining tissues were preserved in RNAsolid (Servicebio, Wuhan, China), snap-frozen in liquid nitrogen, and stored at –80°C.

### 2.5 Serum hormone and phoenixin assays

Serum LH, FSH, testosterone, PNX-14, and PNX-20 were measured using ELISA kits (Wuhan Fine Biotech Co., Ltd.). LH (cat. no. ER1123, detection range: 0.313–20 mIU/mL, sensitivity: 0.188 mIU/mL, intra-assay CV: 5.75%, inter-assay CV: 5.17%); FSH (cat. no. ER0960, detection range: 2.344–150 mIU/mL, sensitivity: 1.406 mIU/mL, intra-assay CV: 5.18%, inter-assay CV: 4.89%); testosterone (cat. no. EU0400-HS, detection range: 31.25–2000 pg/mL, sensitivity: 18.75 pg/mL, intra-assay CV: 5.00%, inter-assay CV: 5.02%); PNX-14 (cat. no. ER1679, detection range: 1.563–100 pg/mL, sensitivity: 0.938 pg/mL, intra-assay CV: 5.09%, inter-assay CV: 5.08%); PNX-20 (cat. no. ER2131, detection range: 7.813–500 pg/mL, sensitivity: 4.688 pg/mL, intra-assay CV: 5.00%, inter-assay CV: 4.98%). Each assay was performed according to the manufacturer’s instructions, with duplicate testing for every sample. The mean value was used for analysis. Detection ranges, sensitivities, and coefficients of variation for each assay were provided by the supplier to ensure reliability.

### 2.6 Histological analysis

Subcutaneous fat was embedded in paraffin and sectioned at 3 μm. Sections were deparaffinized, rehydrated, and stained with hematoxylin and eosin (HE). Adipocyte structure was observed under a light microscope (40×). Cell diameters were measured using Image-Pro Plus 6.0 software, with 10 adipocytes counted in 5 randomly selected fields per group.

### 2.7 Real-time quantitative PCR

Total RNA was extracted from tissues using the FastPure Cell/Tissue Total RNA Isolation Kit V2 (Vazyme Biotech, Nanjing, China). RNA concentration and purity were assessed with a Nanodrop 2000 (A260/A280 ratio 1.8–2.0). cDNA synthesis was performed using the HiScript III RT SuperMix for qPCR (+gDNA wiper) kit. qPCR reactions (20 μL) included 10 μL of 2×ChamQ Universal SYBR Master Mix, 0.4 μL each of forward and reverse primers (10 μM), 2 μL cDNA template, and 7.2 μL nuclease-free water. Cycling conditions were 95 °C for 30 s, followed by 40 cycles of 95 °C for 10 s and 60 °C for 30 s. Genes analyzed included hypothalamic *GnRH, Kiss1, GPR54*, and *Smim20*; pituitary *GnRHr and Smim20*; and *Smim20* in testes and subcutaneous fat. β-actin was used as the internal control. Primer sequences are provided in **Supplementary Table 1**. Relative gene expression levels were calculated using the 2−ΔΔCT method.

### 2.8 Statistical analysis

Data analysis was performed using SPSS 25.0 (IBM, Armonk, NY, USA), and graphs were prepared with GraphPad Prism 8.0. Normality was assessed with the Shapiro–Wilk test (P>0.05 indicating normal distribution). Normally distributed data are presented as mean ± standard deviation ( $\bar{\text{x}}$± s) and compared by independent-sample t-test (two groups) or one-way ANOVA with LSD-t for *post hoc* analysis (multiple groups). Non-normally distributed data are shown as median (P25, P75) and compared with the Mann–Whitney U test (two groups) or Kruskal–Wallis H test (multiple groups). Statistical analysis was performed using two-way ANOVA followed by Tukey’s *post hoc* test when interaction effects were significant, or Bonferroni correction when not significant. Correlations were assessed with Spearman’s rank test. Statistical significance was set at P<0.05 (two-sided).

## 3 Results

### 3.1 High-fat diet induced obesity in male rats

**Figure 1:**
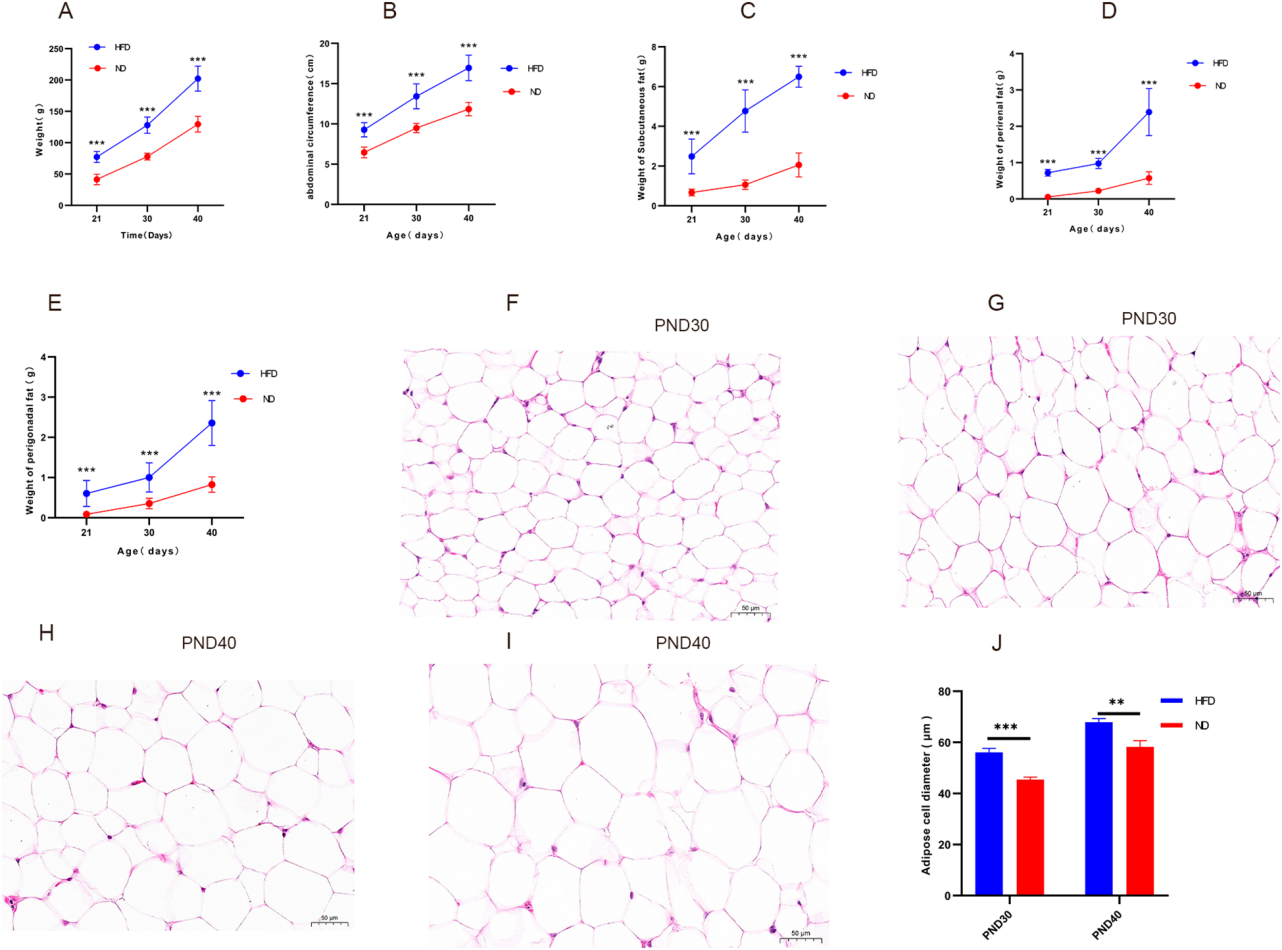
High-fat diet increased body weight, abdominal circumference, fat mass, and promoted adipocyte hypertrophy in male rats Legend: **(A–E)** Changes in body weight, abdominal circumference, mass of subcutaneous fat, perirenal fat, and perigonadal fat in HFD and ND groups. **(F–I)** Representative hematoxylin and eosin (H&E) stained sections (40×) of subcutaneous adipose tissue: **(F)** ND group at PND30, **(G)** HFD group at PND30, **(H)** ND group at PND40, **(I)** HFD group at PND40. Hypertrophic adipocytes are evident in the HFD groups **(G, I)**. **(J)** Mean adipocyte diameter at PND30 and PND40. HFD: high-fat diet; ND: normal diet; PND21: postnatal day 21; PND30: postnatal day 30; PND40: postnatal day 40. Sample sizes: PND30 (HFD and ND, n=9), PND40 (HFD and ND, n=10). **P<0.01, ***P<0.001.

Obesity was defined as body weight ≥20% higher in HFD rats than ND rats at the same time point (30). At weaning (PND21), HFD rats already weighed significantly more than ND rats (77.12 ± 8.83 g vs. 41.31 ± 8.46 g, P<0.001). By prepuberty (PND30) and early puberty (PND40), body weights had risen to 129.57 ± 11.47 g and 203.15 ± 19.57 g, both markedly higher than ND values (76.03 ± 4.85 g and 129.92 ± 14.21 g, P<0.001), with gains exceeding 20% (**Figure 1A**). Abdominal circumference and fat mass: At weaning (PND21), the HFD group showed a significantly greater abdominal circumference (9.27± 0.90 cm) than the ND group (6.46 ± 0.68 cm, P<0.001). The HFD group also had markedly higher subcutaneous, perirenal, and perigonadal fat weights [(2.48 ± 0.88) g, (0.72 ± 0.10) g, and (0.60 ± 0.32) g, respectively] compared with the ND group [(0.67 ± 0.17) g, (0.06 ± 0.02) g, and (0.08 ± 0.04) g; all P<0.001]. At PND30, HFD rats continued to display greater abdominal circumference (13.32 ± 1.73 cm vs. 9.49 ± 0.37 cm, P<0.001) and substantially heavier fat depots (4.5-, 4.3-, and 2.9-fold higher than ND; P<0.001, **Figures 1B–E**). These measures further increased by PND40 (**Figures 1B–E**). Histological analysis confirmed adipocyte hypertrophy, with significantly larger diameters in HFD rats than ND rats (P<0.05, **Figures 1F–I**), Mean adipocyte diameter at PND30 and PND40 (P<0.05, **Figure 1J**). Together, these findings demonstrate successful induction of obesity in juvenile male rats.

### 3.2 High-fat diet accelerated pubertal onset in male rats

**Figure 2:**
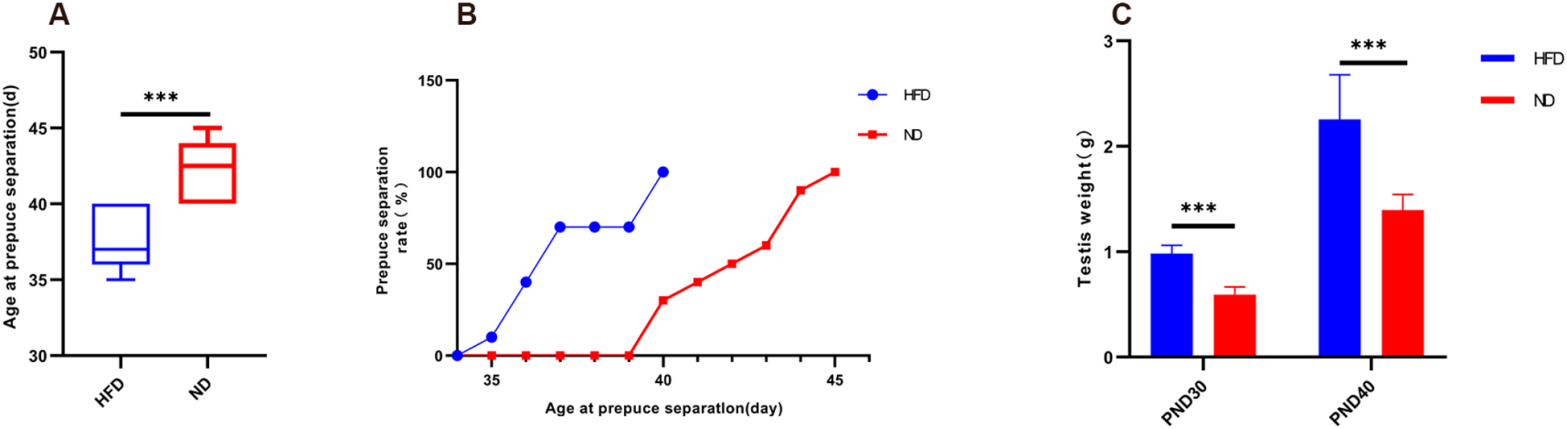
High-fat diet advanced preputial separation and increased testis weight in male rats. **(A)** Boxplot of age at preputial separation. **(B)** Cumulative percentage of preputial separation. **(C)** Testis weight at PND30 and PND40. PND30: postnatal day 30; PND40: postnatal day 40. Sample size: PND30: HFD and ND (n=9); PND40: HFD and ND (n=10). ***P<0.001.

HFD rats reached preputial separation at an earlier age than ND rats (37.40 ± 1.90 vs. 42.30 ± 1.95 days, P<0.001). By PND37, 70% of HFD rats had completed separation, whereas none of the ND rats had (**Figures 2A, B**). Testis weight was also significantly greater in HFD rats at both PND30 (0.98 ± 0.08 g vs. 0.59 ± 0.07 g, P<0.001) and PND40 (2.25 ± 0.42 g vs. 1.40 ± 0.15 g, P<0.001; **Figure 2C**). These results indicate that HFD-induced obesity markedly advanced pubertal onset, accompanied by increased gonadal growth.

### 3.3 High-fat diet increased HPGA gene expression

**Figure 3:**
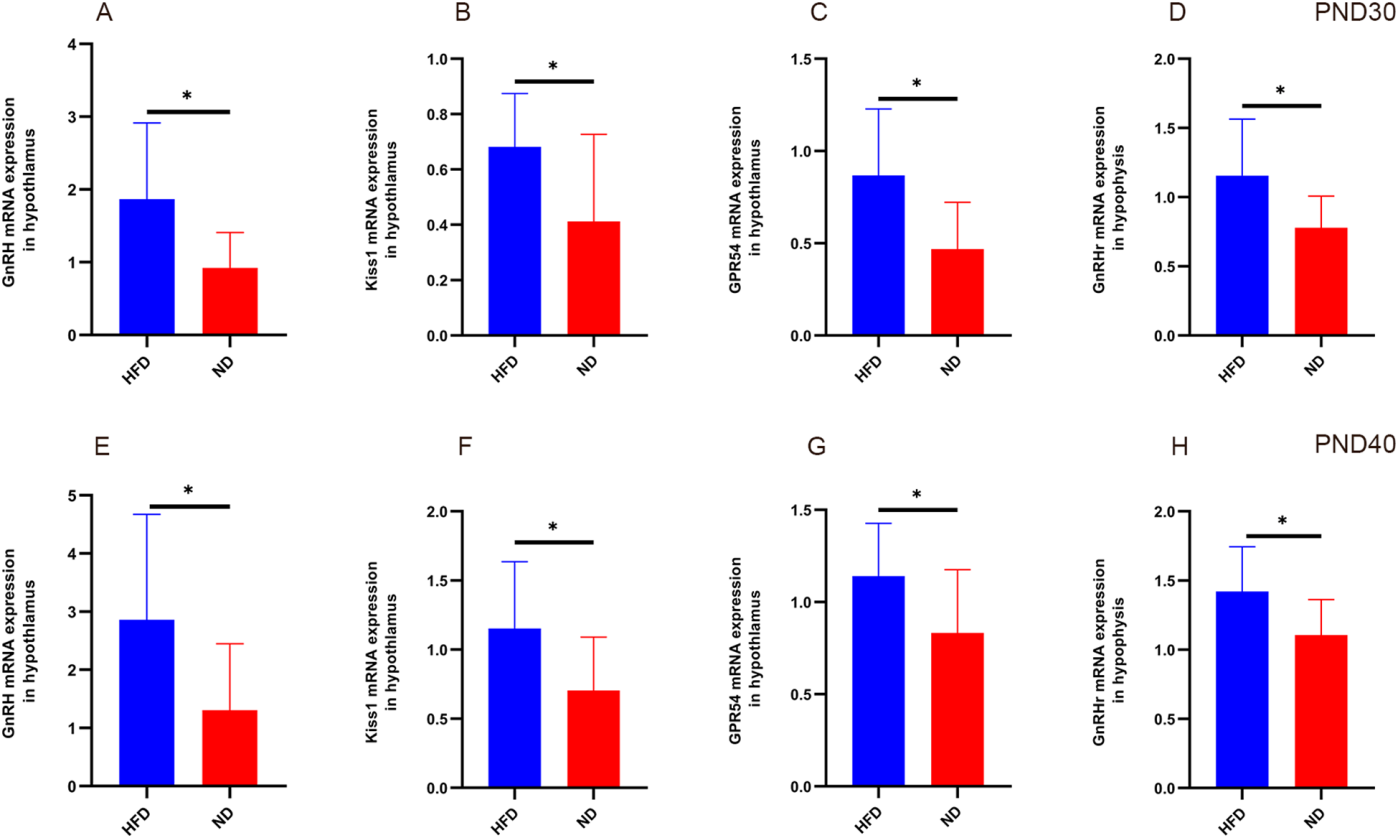
Relative expression of hypothalamic *GnRH, Kiss1, GPR54*, and pituitary *GnRHr* in HFD male rats at different developmental stages. At prepuberty (PND30, panels **A–D**), qPCR analysis showed significantly higher expression of hypothalamic G*nRH, Kiss1, GPR54*, and pituitary *GnRHr* in the HFD group compared with ND (P<0.05). At early puberty (PND40, panels **E–H**), expression of these genes remained elevated in HFD rats (P<0.05). Sample size: PND30: HFD and ND (n=9); PND40: HFD and ND (n=10). Internal control: β-actin. *P<0.05.

At PND30, hypothalamic *GnRH, Kiss1, GPR54*, and pituitary *GnRHr* mRNA levels were significantly higher in HFD rats than ND rats (**Table 1**). At PND40, expression of these genes remained elevated (all P<0.05, **Table 2**). This sustained upregulation suggests activation of HPGA signaling underlies the accelerated pubertal development observed in HFD rats (**Figure 3**).

**Table 1:** mRNA expression levels of hypothalamic *GnRH, Kiss1, and GPR54* genes and pituitary *GnRHr* gene at prepuberty (PND30).

Group	Hypothalamus *GnRH* mRNA	Hypothalamus *Kiss1* mRNA	Hypothalamus *GPR54* mRNA	Hypophysis *GnRHr* mRNA
HFD	1.87 ± 1.05	0.68 ± 0.19	0.87 ± 0.36	1.16 ± 0.41
ND	0.92 ± 0.49	0.41 ± 0.31	0.47 ± 0.25	0.78 ± 0.23
p value	0.025*	0.043*	0.015*	0.029*

HFD, high-fat diet group; ND, normal diet group. *P<0.05.

**Table 2:** mRNA expression levels of hypothalamic *GnRH, Kiss1, and GPR54* genes and pituitary *GnRHr* gene at early puberty (PND40).

Group	Hypothalamus *GnRH* mRNA	Hypothalamus *Kiss1* mRNA	Hypothalamus *GPR54* mRNA	Hypophysis *GnRHr* mRNA
HFD	2.86 ± 1.81	1.15 ± 0.48	1.14 ± 0.29	1.42 ± 0.32
ND	1.30 ± 1.14	0.71 ± 0.39	0.83 ± 0.34	1.1 ± 0.26
p value	0.0341*	0.0343*	0.0420*	0.027*

HFD, high-fat diet group; ND, normal diet group. *P<0.05.

### 3.4 High-fat diet elevated serum sex hormone levels

**Figure 4:**
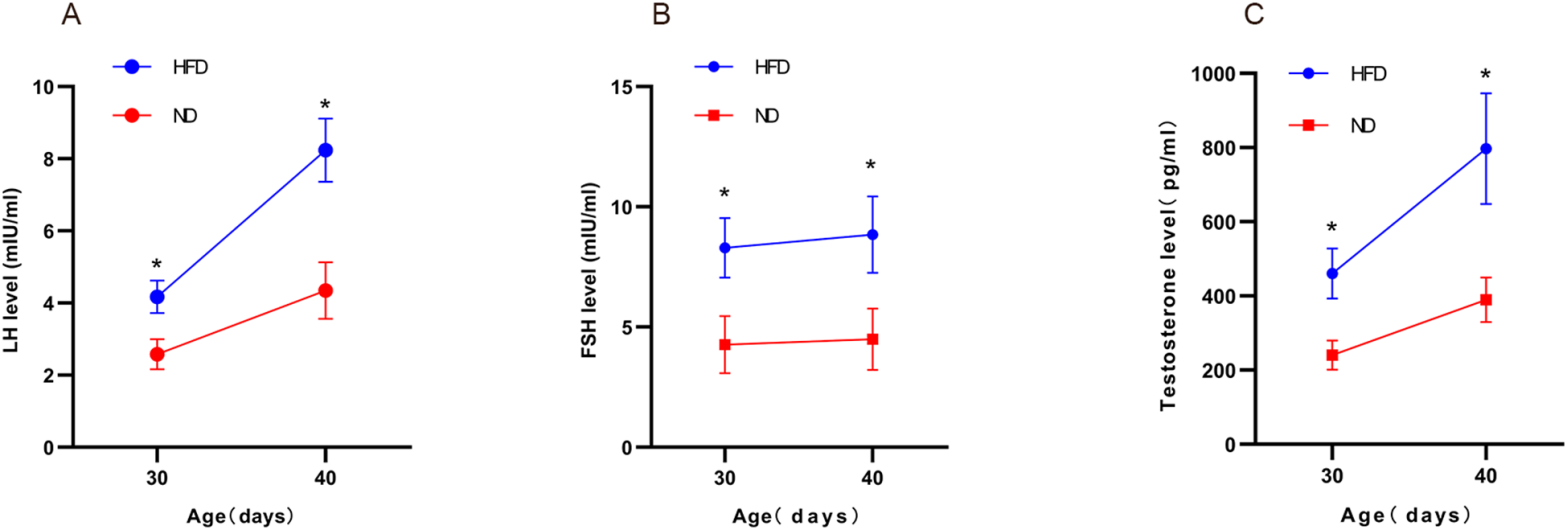
High-fat diet increased serum sex hormone levels in male rats. Serum LH, FSH, and testosterone in male pups under different diets were measured at prepubertal (PND30) and early pubertal (PND40) stages using ELISA **(A–C)**. Sample size: PND30: HFD and ND (n=9); PND40: HFD and ND (n=10). *P<0.05.

ELISA confirmed significantly higher serum LH, FSH, and testosterone in HFD rats compared with ND rats. Two-way ANOVA revealed significant main effects of diet (HFD vs. control) and age (prepubertal vs. early pubertal) on serum LH, FSH, and testosterone levels (p < 0.05), along with significant interaction effects (p < 0.05). The HFD-induced elevation in hormone levels became more pronounced with age. *Post hoc* tests confirmed higher hormone levels in the HFD group at both time points (p < 0.05). In summary, HFD increased serum LH, FSH, and testosterone in male rats, with synergistic effects observed as age progressed. (**Figures 4A–C**). These findings indicate enhanced HPGA activity in HFD rats.

### 3.5 Correlation of obesity and pubertal indices

Spearman analysis revealed that body weight correlated negatively with age at preputial separation but positively with abdominal circumference, serum LH, testosterone, PNX-14, and PNX-20 (**Table 3**). This indicates that obesity-related traits were closely linked to indices of early puberty.

**Table 3:** Correlation between body weight and pubertal development indicators.

Body weigh	Parameters	Prepuce separation time	LH	T	PNX-14	PNX-20	Abdominal perimeter
Prepubertal and early pubertal body weights of male rats	Correlation Coefficient	-0.792	0.783	0.748	0.591	0.552	0.892
	n	20	38	38	38	38	38
	P	<0. 001	<0. 001	<0. 001	<0. 001	<0. 001	<0. 001

LH, luteinizing hormone; FSH, follicle stimulating hormone; T, testosterone.

### 3.6 High-fat diet increased *Smim20* and phoenixin expression

**Figure 5:**
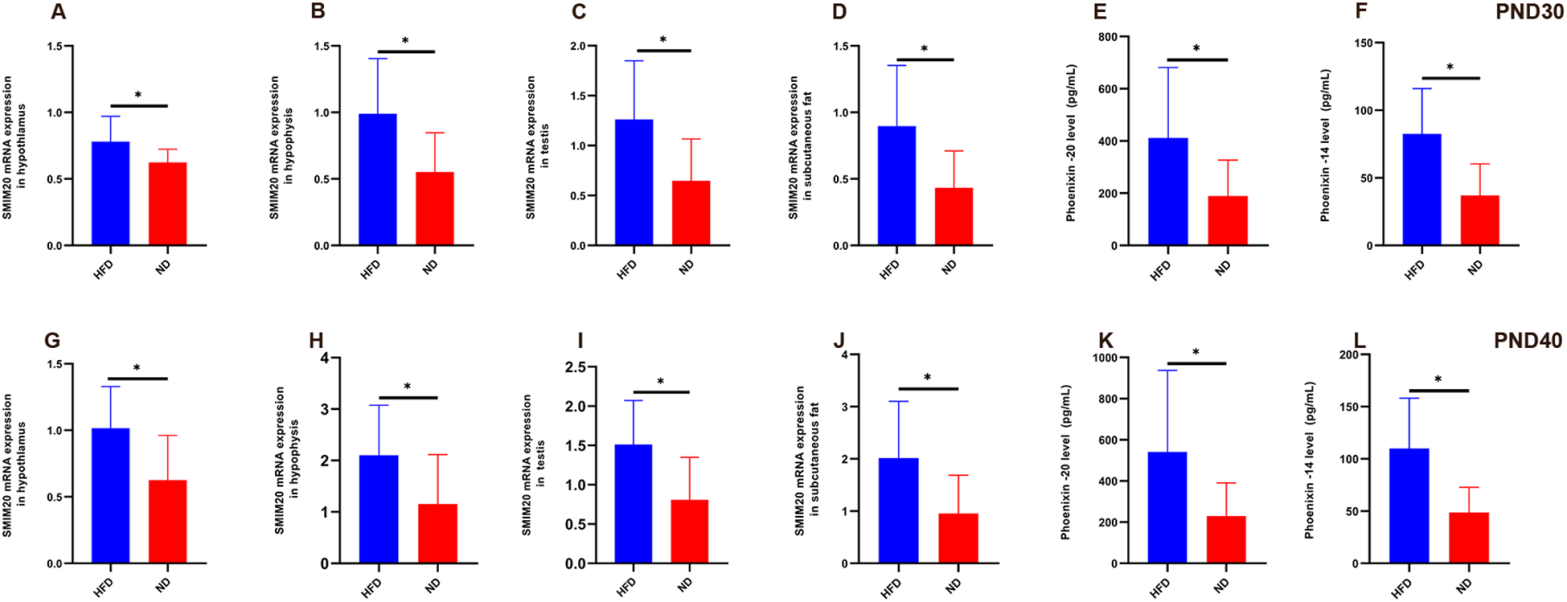
Relative expression of *Smim20* mRNA in hypothalamus, pituitary, testes, and subcutaneous fat, and serum Phoenixin levels in HFD male rats at prepuberty (PND30) and early puberty (PND40) **(A–D)** Relative mRNA expression of Smim20 in the hypothalamus **(A)**, pituitary **(B)**, testes **(C)**, and subcutaneous fat **(D)** at PND30. **(G–J)** Relative mRNA expression of Smim20 in the hypothalamus **(G)**, pituitary **(H)**, testes **(I)**, and subcutaneous fat **(J)** at PND40. **(E, F)** Serum levels of Phoenixin-20 **(E)** and Phoenixin-14 **(F)** measured by ELISA at PND30. **(K, L)** Serum levels of Phoenixin-20 **(K)** and Phoenixin-14 **(L)** measured by ELISA at PND40. Sample size: PND30: HFD and ND (n=9); PND40: HFD and ND (n=10). Note: PND30: postnatal day 30; PND40: postnatal day 40. *P<0.05.

*Smim20* mRNA was significantly upregulated in the hypothalamus, pituitary, testes, and subcutaneous fat of HFD rats at both PND30 and PND40 (all P<0.05; **Tables 4**, **5**, **Figures 5A–D, G–J**).

**Table 4:** mRNA expression levels of Smim20 in the hypothalamus, pituitary, testes, and subcutaneous fat at prepuberty (PND30).

Group	Hypothalamus *Smim20* mRNA	Hypophysis *Smim20* mRNA	Testis *Smim20* mRNA	Subcutaneous fat *Smim20* mRNA
HFD	0.78 ± 0.19	0.99 ± 0.41	1.26 ± 0.59	0.90 ± 0.46
ND	0.62 ± 0.10	0.55 ± 0.29	0.65 ± 0.42	0.43 ± 0.27
P value	0.045*	0.020*	0.022*	0.019*

PND30, postnatal day 30; HFD, high-fat diet group; ND, normal diet group. *P<0.05.

**Table 5:** mRNA expression levels of Smim20 in the hypothalamus, pituitary, testes, and subcutaneous fat at early puberty (PND40).

Group	Hypothalamus *Smim20* mRNA	Hypophysis *Smim20* mRNA	Testis *Smim20* mRNA	Subcutaneous fat *mim20* mRNA
HFD	1.02 ± 0.31	2.1 ± 0.97	1.51 ± 0.56	2.01 ± 1.09
ND	0.63 ± 0.34	1.15 ± 0.96	0.81 ± 0.54	0.96 ± 0.73
P value	0.015*	0.042*	0.011*	0.02*

PND40, postnatal day 40; HFD, high-fat diet group; ND, normal diet group. *P<0.05.

Serum PNX-20 and PNX-14 levels were also higher in HFD rats than ND rats at both stages. At PND30, PNX-20 (411.59 ± 269.21 vs. 188.78 ± 138.20 pg/mL, P = 0.042) and PNX-14 (82.54 ± 33.55 vs. 37.09 ± 23.26 pg/mL, P = 0.004) were significantly elevated (**Figures 5E, F**). At PND40, these differences persisted (PNX-20: 541.42 ± 395.88 vs. 229.33 ± 161.35 pg/mL, P = 0.033; PNX-14: 109.98 ± 48.07 vs. 48.61 ± 24.32 pg/mL, P = 0.002; **Figures 5K, L**). Within the HFD group, serum PNX levels rose from PND30 to PND40, though not significantly (PNX-20, P = 0.42; PNX-14, P = 0.172). Because BMI was higher in HFD rats (53.42 ± 2.25 vs. 46.39 ± 4.01 kg/m²), and PNX-14/20 correlated positively with body weight and BMI, covariance analysis was used to adjust for BMI. Even after adjustment, HFD rats retained significantly higher PNX-20 (479.92 ± 339.13 vs. 210.12 ± 148.11 pg/mL, P<0.001) and PNX-14 (96.98 ± 43.06 vs. 43.16 ± 23.90 pg/mL, P<0.001) levels compared with ND rats.

In summary, HFD feeding elevated *Smim20* expression in multiple tissues and increased circulating PNX-20 and PNX-14 levels. These changes persisted after controlling for body weight, indicating that *Smim20*/PNX contributes independently to the advancement of male puberty (**Figure 5**).

## 4 Discussion

This study used continuous high-fat diet (HFD) intervention from postnatal day 1 (PND1, the onset of lactation) through weaning to successfully establish a prepubertal obesity model in male Sprague–Dawley rats. By PND21, body weight in the HFD group was already significantly higher than in the normal diet (ND) group, and by PND30 and PND40, body weight was more than 20% higher than controls. Abdominal circumference and the weights of subcutaneous, perirenal, and perigonadal fat were also markedly increased (all P<0.001), meeting established criteria for obesity (30). These results are consistent with reports by Ullah et al. (17) and Lainez et al. (31), who validated HFD-induced obesity in female rodents, supporting the suitability of this method for modeling prepubertal obesity in males. Shared features included marked weight gain, fat accumulation, elevated circulating leptin with leptin resistance, and increased hypothalamic Kisspeptin expression—linked to central pubertal activation—along with AgRP/NPY neuron activation that promotes feeding (17, 32–34). However, sex-specific differences were evident: males exhibited greater visceral fat accumulation and hepatic steatosis, whereas females showed more pronounced subcutaneous fat expansion. These differences may partly reflect estrogen’s protective effects on female fat metabolism, contributing to sex-specific insulin sensitivity (35, 36). Thus, although HFD feeding effectively induces obesity in both sexes, the physiological and molecular mechanisms likely differ.

We observed that preputial separation occurred 4.9 days earlier in HFD rats than in ND rats, confirming that obesity induced by HFD accelerates pubertal onset (29). In addition, testis weight and serum levels of LH, FSH, and testosterone were significantly elevated in HFD rats during both prepuberty and early puberty (all P<0.05). These findings parallel clinical observations of elevated free testosterone in obese boys (37), suggesting that HFD promotes pubertal development in males. At the molecular level, hypothalamic *GnRH, Kiss1, GPR54*, and pituitary *GnRHr* mRNA were upregulated in HFD rats at both stages (all P<0.05), in agreement with prior work showing that high-fat, high-sugar diets promote earlier puberty in female rats (38). As an upstream regulator of the HPGA, kisspeptin stimulates GnRH release via binding to GPR54, thereby driving LH, FSH, and steroid hormone production (39, 40). Clinical studies further demonstrate elevated kisspeptin levels in girls with CPP, identifying it as a potential biomarker (21, 41). Our finding that HFD upregulated Kiss1/GPR54 in males is consistent with the female model (17), though the precise mechanisms remain unclear.

Spearman correlation analysis showed that body weight was negatively correlated with age at preputial separation, and positively correlated with abdominal circumference, LH, testosterone, and PNX, confirming that the HFD model appropriately represents the relationship between obesity and puberty. However, a recent study found significantly reduced body weight and delayed puberty in its animal model (42). Taken together, these findings suggest that adequate energy reserves are essential for normal pubertal development, and that both undernutrition and overnutrition can disrupt this process. Other mechanisms, such as p53 overexpression and gut microbiota imbalance, may also contribute to HFD-induced precocious puberty (43, 44). However, the central pathways by which obesity-related metabolic signals trigger premature neuroendocrine activation remain poorly defined.

Given that phoenixin (PNX) is a metabolism-related neuropeptide, we explored its role in linking obesity with precocious puberty. Serum PNX-20 and PNX-14 levels were significantly elevated in HFD rats at both PND30 and PND40 (P<0.05), with the highest levels detected in early puberty (PNX-20: 541.42 ± 395.88 pg/mL; PNX-14: 109.98 ± 48.07 pg/mL). Considering that BMI was higher in HFD rats than in ND rats (53.42 ± 2.25 vs. 46.39 ± 4.01 kg/m²), and that correlation analysis showed positive associations between body weight/BMI and PNX-14/20, these results are consistent with clinical studies reporting a positive correlation between serum PNX and BMI (27, 28). Importantly, after adjusting for BMI, PNX-20 and PNX-14 remained significantly elevated in HFD rats (both P<0.001), suggesting PNX may be an independent factor contributing to early puberty beyond the effects of BMI. Importantly, after adjusting for BMI, both PNX-20 and PNX-14 remained significantly elevated in HFD rats (P < 0.001 for both), suggesting that PNX may be an independent factor contributing to early puberty beyond the effects of BMI. We propose that obesity-related expansion of adipose tissue enhances *Smim20*/PNX expression, thereby accelerating HPGA activation and pubertal onset. PNX is known to regulate adipocyte differentiation, proliferation, and energy balance via the cAMP/Epac pathway (18, 25). Fatty acids can upregulate hypothalamic PNX expression, and excessive adiposity may amplify this effect (18, 45). Furthermore, HFD-induced increases in PNX may exacerbate obesity and insulin resistance (46), and given that insulin resistance is a risk factor for early puberty (47), PNX may indirectly influence pubertal timing through metabolic pathways. Thus, elevated PNX may reflect both metabolic–reproductive cross-talk and direct involvement in HPGA activation.

Our study also demonstrated that *Smim20* mRNA expression in subcutaneous fat increased further in HFD rats during early puberty, consistent with reports of elevated *Smim20* expression in perigonadal fat of heavier rats (20). However, whether PNX derived from peripheral tissues can access the central nervous system to modulate hypothalamic GnRH pulsatility remains uncertain. Importantly, hypothalamic and pituitary *Smim20* mRNA expression also increased in HFD rats, and was positively correlated with serum PNX and *GnRH/GnRHr* expression. These results suggest a central regulatory role for *Smim20*/PNX in pubertal onset. Prior studies show that PNX activates the cAMP/PKA pathway via GPR173, upregulating Kiss1 and stimulating *GnRH* secretion (22, 48). *In vivo*, knockdown of PNX by siRNA delayed estrous cyclicity and reduced *GnRH* expression in female rats (19), while intracerebroventricular injection of PNX increased LH and testosterone levels in male rats (24). Knockdown of GPR173 abolished GnRH-induced LH secretion (23). Previous studies have demonstrated that PNX directly promotes GnRH, LH, FSH, and testosterone secretion (19, 23, 24), consistent with our findings. We also observed a progressive increase in serum PNX from PND30 to PND40 in male rats. Similarly, HFD-induced female rats with precocious puberty showed elevated PNX levels (46). In both sexes, increased PNX levels were associated with pubertal progression, suggesting that PNX may function as a metabolic signal whose continuous elevation facilitates the initiation of puberty.

We further observed that testicular *Smim20* mRNA was upregulated in HFD rats at both PND30 and PND40 (P<0.05), suggesting that *Smim20*/PNX may act at multiple levels—central, gonadal, and peripheral—to regulate male pubertal development. However, the precise mechanisms by which PNX contributes to CPP remain undefined and warrant further *in vivo* and *in vitro* studies.

This study has several limitations. First, although this study examined two developmental time points—prepuberty and early puberty—future longitudinal studies spanning infancy to adulthood are needed to clarify the dynamic and long-term effects of obesity on puberty. In addition, while the present work established the feasibility and reliability of an HFD-induced precocious puberty model in male rats, the underlying molecular mechanisms warrant further investigation. We confirmed positive associations between the model and elevated expression of hypothalamic (GnRH, Kiss1, GPR54), pituitary (GnRHr), and SMIM20 mRNA, but these pathways remain to be explored in detail. Finally, given that modern diets often combine high fat and high sugar, future studies should compare their individual and combined effects on pubertal development.

## 5 Conclusion

This study successfully established a model of HFD-induced prepubertal obesity and central precocious puberty in male rats. We propose that *Smim20*/PNX contributes to early puberty by upregulating mRNA and protein expression in the hypothalamus, pituitary, testes, and adipose tissue, thereby activating the HPGA and enhancing Kisspeptin–GPR54 signaling. This premature activation of GnRH neurons ultimately accelerates pubertal onset. These findings provide experimental evidence for a “metabolic–reproductive axis interaction” in obesity-related precocious puberty and suggest that *Smim20*/PNX may represent a potential target for future interventions.

## Data Availability

The original contributions presented in the study are included in the article/**Supplementary Material**. Further inquiries can be directed to the corresponding author.

## References

[1] Subspecialty Group of Endocrinologic, Hereditary and Metabolic Diseases, the Society of Pediatrics, Chinese Medical Association, Editorial Board, Chinese Journal of Pediatrics . Expert consensus on the diagnosis and treatment of central precocious puberty (2022). Zhonghua er ke za zhi = Chin J Pediatr. (2023) 61:16–22. doi: 10.3760/cma.j.cn112140-20220802-00693, PMID: 36594116

[2] Chen Y Chen J Tang Y Zhang Q Wang Y Li Q . Difference of precocious puberty between before and during the COVID-19 pandemic: A cross-sectional study among shanghai school-aged girls. Front Endocrinol. (2022) 13:839895. doi: 10.3389/fendo.2022.839895, PMID: 35392135 PMC8979840

[3] Bräuner EV Busch AS Eckert-Lind C Koch T Hickey M Juul A . Trends in the incidence of central precocious puberty and normal variant puberty among children in Denmark, 1998 to 2017. JAMA Netw Open. (2020) 3:e2015665. doi: 10.1001/jamanetworkopen.2020.15665, PMID: 33044548 PMC7550972

[4] Shalitin S Gat-Yablonski G . Associations of obesity with linear growth and puberty. Hormone Res Paediatrics. (2022) 95:120–36. doi: 10.1159/000516171, PMID: 34130293

[5] Huang A Roth CL . The link between obesity and puberty: what is new? Curr Opin Pediatr. (2021) 33:449–57. doi: 10.1097/MOP.0000000000001035, PMID: 34173790

[6] Anderson GM Hill JW Kaiser UB Navarro VM Ong KK Perry JRB . Metabolic control of puberty: 60 years in the footsteps of Kennedy and Mitra's seminal work. Nat Rev Endocrinol. (2024) 20:111–23. doi: 10.1038/s41574-023-00919-z, PMID: 38049643 PMC10843588

[7] Qing Y Jamal MA Shi D Zhao S Xu K Jiao D . Delayed body development with reduced triglycerides levels in leptin transgenic pigs. Transgenic Res. (2022) 31:59–72. doi: 10.1007/s11248-021-00288-1, PMID: 34741281

[8] Reinehr T Roth CL . Is there a causal relationship between obesity and puberty? Lancet Child Adolesc Health. (2019) 3:44–54. doi: 10.1016/S2352-4642(18)30306-7, PMID: 30446301

[9] Yaisilp P Numsriskulrat N Sahakitrungruang T . Clinical and epidemiological insights into early puberty in Thai girls: a 5-year study. Ann Pediatr Endocrinol Metab. (2025) 30:17–24. doi: 10.6065/apem.2448112.056, PMID: 40049671 PMC11917400

[10] Huang JS Gao C Xiao WQ Zhang XY Zhong XW Qin YQ . Association of childhood obesity with pubertal development in boys: A systematic review and meta-analysis. Obes Rev. (2025) 26:e13869. doi: 10.1111/obr.13869, PMID: 39567861

[11] Pereira A Busch AS Solares F Baier I Corvalan C Mericq V . Total and central adiposity are associated with age at gonadarche and incidence of precocious gonadarche in boys. J Clin Endocrinol Metab. (2021) 106:1352–61. doi: 10.1210/clinem/dgab064, PMID: 33539513

[12] O'Keeffe LM Frysz M Bell JA Howe LD Fraser A . Puberty timing and adiposity change across childhood and adolescence: disentangling cause and consequence. Hum Reprod. (2020) 35:2784–92. doi: 10.1093/humrep/deaa213, PMID: 33242326 PMC7744159

[13] Calcaterra V Tiranini L Magenes VC Rossi V Cucinella L Nappi RE . Impact of obesity on pubertal timing and male fertility. J Clin Med. (2025) 14:783. doi: 10.3390/jcm14030783, PMID: 39941454 PMC11818283

[14] Song Y Kong Y Xie X Wang Y Wang N . Association between precocious puberty and obesity risk in children: a systematic review and meta-analysis. Front Pediatr. (2023) 11:1226933. doi: 10.3389/fped.2023.1226933, PMID: 37635793 PMC10456873

[15] Cassio A Marescotti G Aversa T Salerno M Tornese G Stancampiano M . Central precocious puberty in italian boys: data from a large nationwide cohort. J Clin Endocrinol Metab. (2024) 109:2061–70. doi: 10.1210/clinem/dgae035, PMID: 38308814 PMC11244209

[16] Manfredi-Lozano M Roa J Tena-Sempere M . Connecting metabolism and gonadal function: Novel central neuropeptide pathways involved in the metabolic control of puberty and fertility. Front Neuroendocrinol. (2018) 48:37–49. doi: 10.1016/j.yfrne.2017.07.008, PMID: 28754629

[17] Ullah R Su Y Shen Y Li C Xu X Zhang J . Postnatal feeding with high-fat diet induces obesity and precocious puberty in C57BL/6J mouse pups: a novel model of obesity and puberty. Front Med. (2017) 11:266–76. doi: 10.1007/s11684-017-0530-y, PMID: 28500430

[18] Muzammil AN Barathan M Yazid MD Sulaiman N Makpol S Mohamed Ibrahim N . A systematic scoping review of the multifaceted role of phoenixin in metabolism: insights from *in vitro* and *in vivo* studies. Front Endocrinol. (2024) 15:1406531. doi: 10.3389/fendo.2024.1406531, PMID: 39398330 PMC11466790

[19] Yosten GL Lyu RM Hsueh AJ Avsian-Kretchmer O Chang JK Tullock CW . A novel reproductive peptide, phoenixin. J Neuroendocrinol. (2013) 25:206–15. doi: 10.1111/j.1365-2826.2012.02381.x, PMID: 22963497 PMC3556183

[20] Kalamon N Błaszczyk K Szlaga A Billert M Skrzypski M Pawlicki P . Levels of the neuropeptide phoenixin-14 and its receptor GRP173 in the hypothalamus, ovary and periovarian adipose tissue in rat model of polycystic ovary syndrome. Biochem Biophys Res Commun. (2020) 528:628–35. doi: 10.1016/j.bbrc.2020.05.101, PMID: 32505354

[21] Li M Chen Y Liao B Tang J Zhong J Lan D . The role of kisspeptin and MKRN3 in the diagnosis of central precocious puberty in girls. Endocrine Connect. (2021) 10:1147–54. doi: 10.1530/EC-21-0182, PMID: 34414898 PMC8494402

[22] Ke R Ma X Lee LTO . Understanding the functions of kisspeptin and kisspeptin receptor (Kiss1R) from clinical case studies. Peptides. (2019) 120:170019. doi: 10.1530/EC-21-0182, PMID: 30339828

[23] Stein LM Tullock CW Mathews SK Garcia-Galiano D Elias CF Samson WK . Hypothalamic action of phoenixin to control reproductive hormone secretion in females: importance of the orphan G protein-coupled receptor Gpr173. Am J Physiol Regulatory Integr Comp Physiol. (2016) 311:R489–96. doi: 10.1152/ajpregu.00191.2016, PMID: 27440717 PMC5142227

[24] Guvenc G Altinbas B Kasikci E Ozyurt E Bas A Udum D . Contingent role of phoenixin and nesfatin-1 on secretions of the male reproductive hormones. Andrologia. (2019) 51:e13410. doi: 10.1111/and.13410, PMID: 31637758

[25] Billert M Wojciechowicz T Jasaszwili M Szczepankiewicz D Waśko J Kaźmierczak S . Phoenixin-14 stimulates differentiation of 3T3-L1 preadipocytes via cAMP/Epac-dependent mechanism. Biochim Biophys Acta (BBA) - Mol Cell Biol Lipids. (2018) 1863:1449–57. doi: 10.1016/j.bbalip.2018.09.006, PMID: 30251651

[26] Nguyen XP Nakamura T Osuka S Bayasula B Nakanishi N Kasahara Y . Effect of the neuropeptide phoenixin and its receptor GPR173 during folliculogenesis. Reproduction. (2019) 158:25–34. doi: 10.1530/REP-19-0025, PMID: 30933929

[27] Xie T Qin W Zeng D Wang R Chen Y Lan D . Elevated serum phoenixin levels in boys with central precocious puberty are positively correlated with BMI. Endocrine Connect. (2025) 14:e250358. doi: 10.1530/EC-25-0358, PMID: 40823903 PMC12400499

[28] Yang Y Sun J Yang S Li S Zhang J Zhu F . Serum phoenixin levels and their diagnostic significance in girls with precocious puberty. J Endocrine Soc. (2025) 9:bvaf065. doi: 10.1210/jendso/bvaf065, PMID: 40421429 PMC12105469

[29] Uğurlu AK Bideci A Demirel AM Kaplanoğlu GT Dayanır D Gülbahar Ö . Is blue light exposure a cause of precocious puberty in male rats? Front Endocrinol. (2023) 14:1190445. doi: 10.3389/fendo.2023.1190445, PMID: 37409230 PMC10319012

[30] Dong M Liang X Zhu T Xu T Xie L Feng Y . Reoxygenation mitigates intermittent hypoxia-induced systemic inflammation and gut microbiota dysbiosis in high-fat diet-induced obese rats. Nat Sci Sleep. (2024) 16:517–30. doi: 10.2147/NSS.S454297, PMID: 38812701 PMC11135559

[31] Lainez NM Jonak CR Nair MG Ethell IM Wilson EH Carson MJ . Diet-induced obesity elicits macrophage infiltration and reduction in spine density in the hypothalami of male but not female mice. Front Immunol. (2018) 9:1992. doi: 10.3389/fimmu.2018.01992, PMID: 30254630 PMC6141693

[32] Haque N Tischkau SA . Sexual dimorphism in adipose-hypothalamic crosstalk and the contribution of aryl hydrocarbon receptor to regulate energy homeostasis. Int J Mol Sci. (2022) 23:7679. doi: 10.3390/ijms23147679, PMID: 35887027 PMC9322714

[33] Guzzardi MA Guiducci L Campani D La Rosa F Cacciato Insilla A Bartoli A . Leptin resistance before and after obesity: evidence that tissue glucose uptake underlies adipocyte enlargement and liver steatosis/steatohepatitis in Zucker rats from early-life stages. Int J Obes. (2022) 46:50–8. doi: 10.1038/s41366-021-00941-z, PMID: 34489524

[34] Vohra MS Benchoula K Serpell CJ Hwa WE . AgRP/NPY and POMC neurons in the arcuate nucleus and their potential role in treatment of obesity. Eur J Pharmacol. (2022) 915:174611. doi: 10.1016/j.ejphar.2021.174611, PMID: 34798121

[35] Escalona R Larqué C Cortes D Vilchis R Granados-Delgado E Sánchez A . High-fat diet impairs glucose homeostasis by increased p16 beta-cell expression and alters glucose homeostasis of the progeny in a parental-sex dependent manner. Front Endocrinol. (2023) 14:1246194. doi: 10.3389/fendo.2023.1246194, PMID: 37876538 PMC10591070

[36] Comas F Díaz-Trelles R Gavaldà-Navarro A Milbank E Dragano N Morón-Ros S . Downregulation of peripheral lipopolysaccharide binding protein impacts on perigonadal adipose tissue only in female mice. Biomed Pharmacother = Biomed Pharmacother. (2022) 151:113156. doi: 10.1016/j.biopha.2022.113156, PMID: 35643066

[37] Vandewalle S De Schepper J Kaufman JM . Androgens and obesity in male adolescents. Curr Opin Endocrinol Diabetes Obes. (2015) 22:230–7. doi: 10.1097/MED.0000000000000160, PMID: 25871956

[38] Huang XY Chen JX Ren Y Luo HL Xiang W He XJ . Postnatal feeding with high-fat combined with high-glucose diet induces precocious puberty in Sprague–Dawley rat pups. Biochem Biophys Res Commun. (2024) 693:149199. doi: 10.1016/j.bbrc.2023.149199, PMID: 38118311

[39] Shim YS Lee HS Hwang JS . Genetic factors in precocious puberty. Clin Exp Pediatr. (2022) 65:172–81. doi: 10.3345/cep.2021.00521, PMID: 34665958 PMC8990949

[40] Trevisan CM Montagna E De Oliveira R Christofolini DM Barbosa CP Crandall KA . Kisspeptin/GPR54 system: what do we know about its role in human reproduction? Cell Physiol Biochem. (2018) 49:1259–76. doi: 10.1159/000493406, PMID: 30205368

[41] Vuralli D Ciftci N Demirbilek H . Serum kisspeptin, neurokinin B and inhibin B levels can be used as alternative parameters to distinguish idiopathic CPP from premature thelarche in the early stages of puberty. Clin Endocrinol. (2023) 98:788–95. doi: 10.1111/cen.14906, PMID: 36879296

[42] Jamal MA Cheng Y Jiao D Cheng W Zou D Wang X . Unraveling the impact of hyperleptinemia on female reproduction: insights from transgenic pig model. Biol Res. (2024) 57:60. doi: 10.1186/s40659-024-00545-7, PMID: 39227998 PMC11373500

[43] Chen T Chen C Wu H Chen X Xie R Wang F . Overexpression of p53 accelerates puberty in high-fat diet-fed mice through Lin28/let-7 system. Exp Biol Med. (2021) 246:66–71. doi: 10.1177/1535370220961320, PMID: 32996351 PMC7797992

[44] Bo T Liu M Tang L Lv J Wen J Wang D . Effects of high-fat diet during childhood on precocious puberty and gut microbiota in mice. Front Microbiol. (2022) 13:930747. doi: 10.3389/fmicb.2022.930747, PMID: 35910597 PMC9329965

[45] McIlwraith EK Loganathan N Belsham DD . Phoenixin expression is regulated by the fatty acids palmitate, docosahexaenoic acid and oleate, and the endocrine disrupting chemical bisphenol A in immortalized hypothalamic neurons. Front Neurosci. (2018) 12:838. doi: 10.3389/fnins.2018.00838, PMID: 30524225 PMC6262291

[46] Valsamakis G Arapaki A Balafoutas D Charmandari E Vlahos NF . Diet-induced hypothalamic inflammation, phoenixin, and subsequent precocious puberty. Nutrients. (2021) 13:3460. doi: 10.3390/nu13103460, PMID: 34684462 PMC8540795

[47] Sun Y Liu H Mu C Liu P Hao C Xin Y . Early puberty: a review on its role as a risk factor for metabolic and mental disorders. Front Pediatr. (2024) 12:1326864. doi: 10.3389/fped.2024.1326864, PMID: 39328587 PMC11424421

[48] Treen AK Luo V Belsham DD . Belsham, phoenixin activates immortalized gnRH and kisspeptin neurons through the novel receptor GPR173. Mol Endocrinol. (2016) 30:872–88. doi: 10.1210/me.2016-1039, PMID: 27268078 PMC5414621

