# Supplementary material for: High-fat diet-induced obesity accelerates puberty in male rats through SMIM20/phoenixin upregulation
Source: Front Endocrinol (Lausanne). 2025 Nov 21;16:1711374. doi: 10.3389/fendo.2025.1711374 (PMC12679827; doi:10.3389/fendo.2025.1711374)
Supplement: Supplementary file 1 [file Table1.docx]

Supplementary Material

**Table 1 Primer sequences used in this study**

| genes |  | Sequences（5' to 3'） |
| --- | --- | --- |
| *GnRH* | F | AGGAGCTCTGGAACGTCTGAT |
|  | R | AGCGTCAATGTCACACTCGG |
| *GnRHr* | F | GCTGCCTGTTCATCATCCCTCTTC |
|  | R | GCTGTAGTTTGCGTGGGTCCTG |
| *Smim20* | F | GCGCTCATATTCGGAGGCT |
|  | R | AGCCTGCTCCTTCTGGTATT |
| *KISS 1* | F | ATGATCTCGCTGGCTTCTTGG |
|  | R | GGTTCAGGGTTCACCACA GG |
| *GPR54* | F | TTTCCTTCTGTGCTGCGTACC |
|  | R | CGAGACCTGCTGGATGTAGTTG |
| *β－actin* | F | TGTCACCAACTGGGACTATA |
|  | R | TCTCAGCTGTGGTGGTGAAG |

Note: F: Forward Sequence；R：Reverse Sequence
